# A simple method for restoring the femoral head center in hip arthroplasty: a 3-dimensional analysis in the Chinese population

**DOI:** 10.1186/s12891-022-05901-w

**Published:** 2022-11-15

**Authors:** Zui Tian, Xingjia Mao, Zhenzhong Gao, Bohong Chen, Zehua Wang, Zhiwen Yin, Zijian Guo, Ze Gao, Chuan Xiang

**Affiliations:** 1 Department of Orthopedics, Second Hospital of Shanxi Medical University, Taiyuan, China; 2 Taiyuan Central Hospital of Shanxi Medical University, Taiyuan, China; 3 The First Affiliated Hospital Of Xi’an Jiaotong University, Xi’an, China

**Keywords:** Femoral head center, Greater trochanter, Lesser trochanter, Hip arthroplasty

## Abstract

**Background:**

Various authors have successfully demonstrated that the distance from the greater trochanter to the femoral head center (GTFHC) and the distance from the lesser trochanter to the femoral head center (LTFHC) can be used as parameters to determine the recovery of the femoral head center (FHC) during hip arthroplasty. It is necessary to undertake an anatomical study concerning the correlations between the greater trochanter (GT), the lesser trochanter (LT), and the FHC using data obtained from the 3D-CT reconstruction method.

**Methods:**

The study comprised 293 patients (151 males and 142 females), with an average age of 65.06 years. The femoral head diameter(FHD), the linear distance from FHC to GT (GTFHC), and the linear distance from FHC to LT(LTFHC) were all measured and recorded data. The correlation between FHD with LTFHC and GTFHC was assessed using Pearson correlation coefficients, and the ratio of LTFHC and GTFHC to FHD was calculated from this ratio. All measured parameters were compared between the left and right sides and the sexes of the participants.

**Results:**

The average ratios of GTFHC/FHD and LTFHC/FHD were 0.99 and 0.95, respectively .96% of the LTFHC had absolute lateral differences of < 4 mm . 92% of the GTFHC had absolute lateral differences of < 4 mm.

**Conclusion:**

LTFHC and GTFHC are reliable reference parameters for preoperative planning and reconstruction of FHC of hip arthroplasty. The ratio displayed in this research may yield insight into a practical and straightforward method for orthopedic surgeons to perform hip arthroplasty in patients with femoral neck fractures. Ratios from studies based on the same race may be desirable for future work.

## Introduction

With the progressive aging of the population, the incidence of hip fractures is increasing worldwide [1]. Hip arthroplasty is an effective therapy for treating femoral neck fractures, which can rapidly reconstruct hip function and avoid a series of complications such as infection and thrombosis caused by long-term bed rest, and can effectively reduce the morbidity and mortality rate while improving patients' quality of life [2].

The femoral head center (FHC) is biomechanically significant and is an important reference point for considering prosthesis placement in hip arthroplasty [3, 4]. The inappropriate position of the FHC after surgery can lead to complications such as altered lower limb force lines, uncoordinated gait, sciatica, and abduction weakness [5–7]. These complications can rapidly develop into unsatisfactory postoperative outcomes for patients.Therefore,accurate and reproducible identification of the FHC is necessary for the surgeon to restore normal hip biomechanics. As a result, the surgeon needs to be able to accurately and consistently identify the HJC in order to improve preoperative planning and restore normal hip kinematics.

Various authors have demonstrated that the linear distance from FHC to GT (GTFHC) and the linear distance from FHC to LT(LTFHC) can be successfully used as parameters for the recovery of the FHC in hip arthroplasty [8–11]. However, the majority of studies on the parameters of LTFHC are based on Western populations and not on Asian populations [8–11]. There are discrete variations between Asian and Western skeletons. To our knowledge, the study on parameter GTFHC was based on two-dimensional images(radiographs, which are susceptible to projection errors in the three-dimensional geometry of the proximal femur), which is potentially reduced in 3D scans. And few studies have investigated parametric LTFHC in Chinese populations. And Under this rationale, it is necessary to perform an anatomical study of the correlations between the GT, the LT, and the FHC in the Chinese population based on the method of 3D-CT reconstruction. This study aimed to: (1) establish a left–right difference analysis of LTFHC and GTFHC. (2) investigate the correlation between the GT, the LT, and the FHC in the Chinese population. (3) compare the differences between our study and the earlier research.

## Patients and methods

This study was approved by the Ethics Committee of the Second Hospital of Shanxi Medical University.

### Participants

This study selected 293 CT images of patients attending the Second Hospital of Shanxi Medical University from January 2020 to January 2022. 151 males and 142 females participated in this study, with a mean age of 65.06 years. Participants’ minimum and maximum ages were 21 and 95, respectively.

#### Inclusion criteria

1. patients were ≥ 20 years of age; (2) complete bilateral hip CT data were available.

#### Exclusion criteria

- (1) advanced degenerative changes of the hip; (2) history of rheumatic immune system diseases; (3) previous surgical history, bone disease (metabolic disease or malignancy), or trauma of the lower extremity (4) developmental dysplasia of the hip; (5) morphology of the greater trochanter is flat or false double apical; (6) poorly documented individuals.

### Imaging procedures

The equipment used to collect the data was a German Siemens 64-row double spiral CT from the imaging department of the Second Hospital of Shanxi Medical University. The patients were scanned electronically by computed tomography. Scanning conditions included a scan voltage of 120 kV, current of 210 mAs, a layer thickness of 0.699 mm, window width of 2500 Hu, bed position of 100 Hu, and a scan matrix of 512 × 512. Image processing was performed with MIMICS 20.0 and 3-Matic 9.0 (Materialise’s Interactive Medical lmage Control System, Materialise*, Materialise HQ) software.

### 3D reconstruction

The DICOM images were imported into Mimics 20.0 software (Materialise, Belgium) for the following, tuning contrast, threshold setting, region growing functionality, and 3D reconstruction to generate the 3D models.

### Identification of Bony Landmarks

In three dimensions, the point chosen to represent the LT was identified. This position was chosen in the proximal–distal and mediolateral directions, at the intersection of the proximal aspect of the LT with the proximal femoral metaphysis. The point is located at the center of this junction in the anteroposterior direction.

A single sphere containing at least 80% of the entire subchondral surface of the femoral head is then selected and used as the basis for calculating the best-fit individual spheres. The point represents FHC is the geometric center of this sphere.

The point represents GT is the the apex of the greater trochanter.

### 3D Measurement

**Fig. 1:**
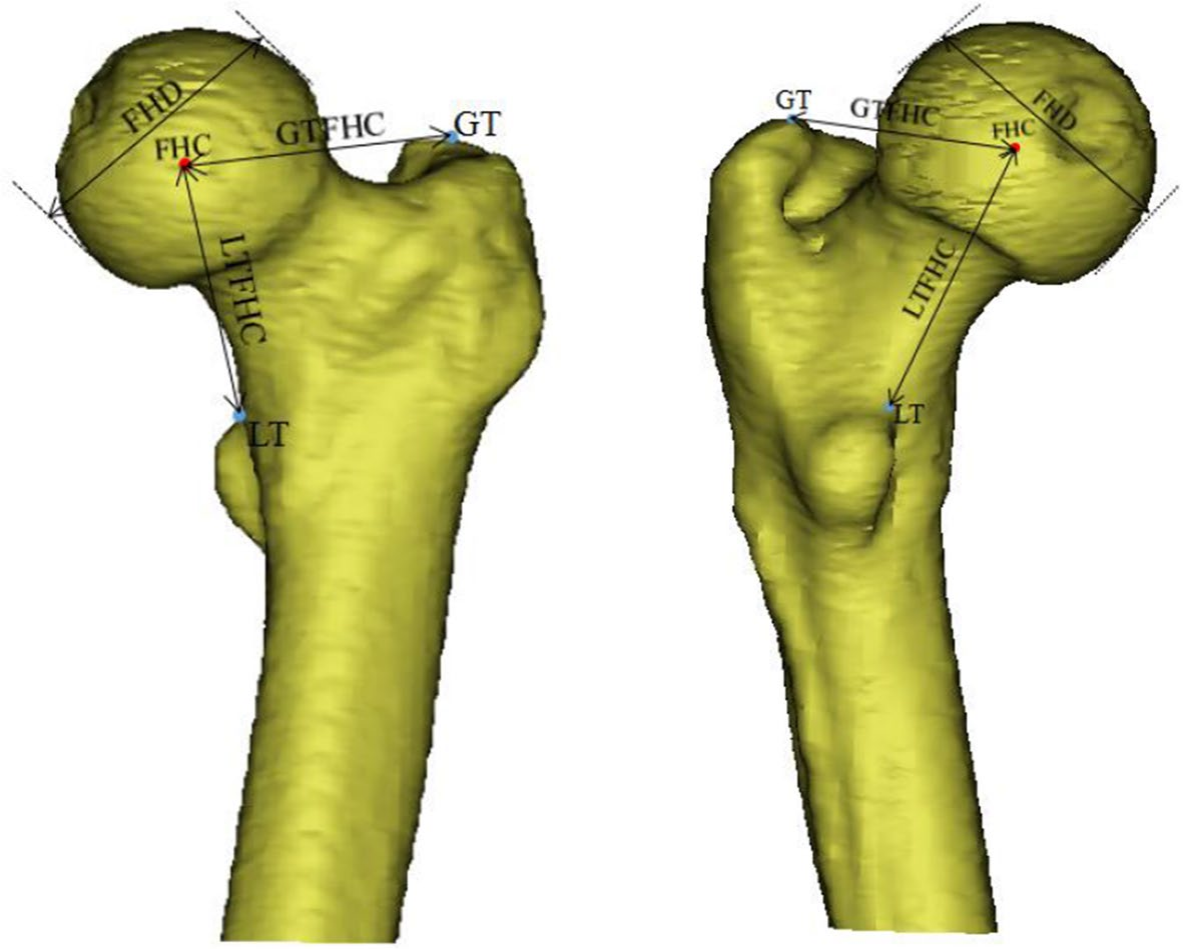
FHD, femoral head diameter; LT, lesser trochanter; LTFHC, linear distance from the FHC to the LT; GT, greater trochanter; GTFHC, linear distance from the FHC to the GT

The 3D model is imported into 3-Matic research 11.0 (Materialise, Belgium) for 3D measurement. The femoral head diameter(FHD), the linear distance from FHC to GT (GTFHC), and the linear distance from FHC to LT (LTFHC) were all measured and recorded, as shown in Fig. 1. We randomly selected 50 samples to measure all parameters twice at an interval of 5 days by two observers to determine intra- and inter-observer measurement reliability.

### Statistics

Intra-and inter-observer measurement reliabilities were analyzed using intraclass correlation coefficient (ICC).The mean and standard deviation (SDS) of all parameters were determined, and the data were tested for normality using the Kolmogorov–Smirnov test. All comparisons between genders were performed using two independent samples. These tests were the t-test (normally distributed parameters) or the Mann–Whitney U-test (non-normally distributed parameters). A two-tailed paired t-test was applied to assess the variation between the left and right sides. Following this, Pearson correlation coefficient and linear regression analyses were used to evaluate and identify the correlation of FHD with GTFHC and LTFHC. Statistical analysis was performed using IBM SPSS Statistics 23.0 (IBM, Armonk, NY, USA).

## Results

### Intra-observer and inter-observer variability

All ICC values were above 0.91, indicating a very high intra- and inter-observer reliability (Table 1).

**Table 1:** Intra- and inter-observer reliability

	Intra- observer reliability		Inter-observer reliability	
	ICC	*P*	ICC	*P*
FHD(mm)	0.98	<0.01	0.97	< .001
LTFHC(mm)	0.95	<0.01	0.96	< .001
GTFHC(mm)	0.92	<0.01	0.91	< 0.01

*Abbreviations*: *FHD* Femoral head diameter, *LTFHC* Linear distance from the FHC to the LT, *GTFHC* Linear distance from the FHC to the GT.

### Measured distance

The mean FHD was 47.26 ± 3.41 mm, the distance LTFHC was 45.08 ± 4.11 mm, and the distance GTFHC was 46.83 ± 4.34 mm. The mean LTFHC/FHD was 0.95 ± 0.06, with a correlation coefficient (r) of 0.650 (*p* < 0.01). The mean GTFHC/FHD was 0.99 ± 0.07, with a correlation coefficient (r) of 0.655 (*p* < 0.01), as shown in Table 2.

### Comparison between left and right sides

The mean absolute value of the lateral difference for LTFHC was 1.41 ± 1.18 mm, the mean lateral difference for LTFHC was 0.09 ± 1.83 mm with a correlation coefficient of *r* = 0.90 (*p* < 0.01). The mean absolute value of the lateral difference for GTFHC was 1.81 ± 1.41 mm, the mean lateral difference for GTFHC was 0.17 ± 2.29 mm with a correlation coefficient of *r* = 0.861 (*p* < 0.01). Overall, 76% of the LTFHC had absolute lateral differences of < 2 mm, where 20% of the LTFHC were between 2 and 4 mm, and 4% of the LTFHC were > 4 mm. 63% of the GTFHC had absolute lateral differences of < 2 mm, 29% of the GTFHC were between 2 and 4 mm, and 8% of the GTFHC were > 4 mm. Further information regarding differences between the left and right sides of the femoral anatomy is listed in Table 3.

**Table 2:** Measurement results and differences between males and females

Variables	Total	Male	Female	P
FHD(mm)	47.26 ± 3.41	49.7074 ± 2.32	44.67 ± 2.26	< .001
LTFHC(mm)	45.08 ± 4.11	46.8886 ± 3.61	43.15 ± 3.70	< .001
LTFHC/FHD	0.95 ± 0.06	0.94 ± 0.06	0.96 ± 0.07	< 0.01
R(LTFHC-FHD)	0.650	0.497	0.561	
GTFHC(mm)	46.83 ± 4.34	48.8569 ± 3.6849	44.68 ± 3.94	< 0.01
GTFHC/FHD	0.99 ± 0.07	0.98 ± 0.06	1.00 ± 0.08	< 0.01
R(GTFHC-FHD)	0.655	0.520	0.494	

*Abbreviations*: *FHD* Femoral head diameter, *LT* Lesser trochanter, *LTFHC* Linear distance from the FHC to the LT, *GT* Greater trochanter, *GTFHC* Linear distance from the FHC to the GT.P was compared between genders

### Comparison between males and females

The mean FHD was 49.71 ± 2.71 mm in males and 46.88 ± 3.30 mm in females; the mean FHD was 6% higher in the male population than in the female population. LTFHC/FHD was 0.94 ± 0.07 in males and 0.96 ± 0.07 in females. GTFHC/FHD was 0.98 ± 0.07 in males and 1.00 ± 0.07 in females, and the difference between the male and female populations was determined to be statistically significant. Additional information regarding the gender differences in femoral anatomy is listed in Table 2.

**Table 3:** Measurement differences between left and right sides

Variables	Right	Left	*P*
FHD (mm)	47.40± 3.44	47.13 ± 3.37	< 0.01
LTFHC (mm)	45.03± 4.08	45.12± 4.12	0.410
LTFHC/FHD	0.95 ± 0.06	0.96 ± 0.06	< 0.01
R(LTFHC-FHD)	0.654	0.649	
GTFHC (mm)	46.74 ± 4.40	46.92 ± 4.29	0.196
GTFHC/FHD	0.99 ± 0.07	1.00 ± 0.07	< 0.01
R(GTFHC-FHD)	0.641	0.672	

*Abbreviations*: *FHD* Femoral head diameter, *LT* Lesser trochanter, *LTFHC* Linear distance from the FHC to the LT, *GT* Greater trochanter, *GTFHC* Linear distance from the FHC to the GT

### Pearson correlation coefficient and linear regression analysis

**Fig. 2:**
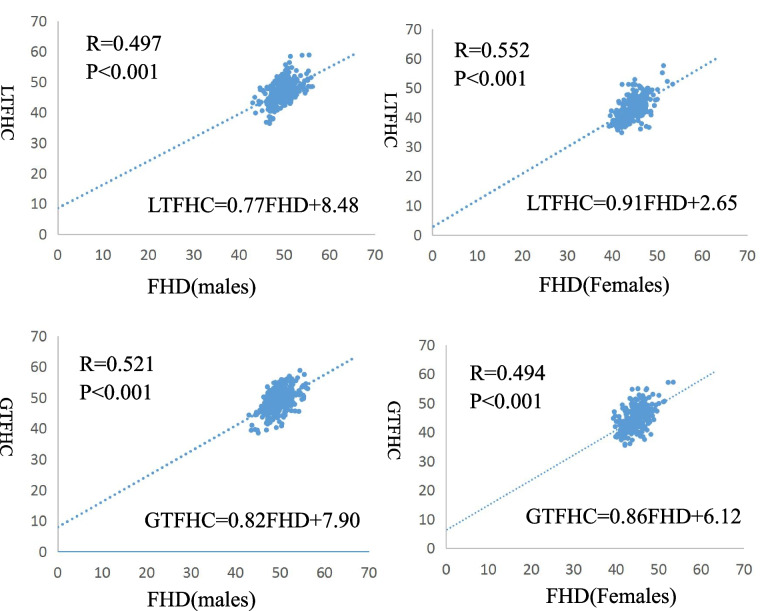
Correlation between FHD and the LTFHC、GTFHC when males and females are considered individually

The relationship between LTFHC and FHD for males being *R* = 0.497, *P* ≤ 0.001 and LTFHC = 0.77FHD + 8.48 and for females the results were *R* = 0.552, *P* < 0.001 and LTFHC = 0.91 FHD + 2.65, respectively. The relationship between GTFHC and FHD for males was *R* = 0.521, *P* ≤ 0.001 and GTFHC = 0.82FHD + 7.90 and for females, *R* = 0.494, *P* < 0.001 and GTFHC = 0.86FHD + 6.12,respectively, as shown in Fig. 2.

### Differences between this study and the previous literature

**Fig. 3:**
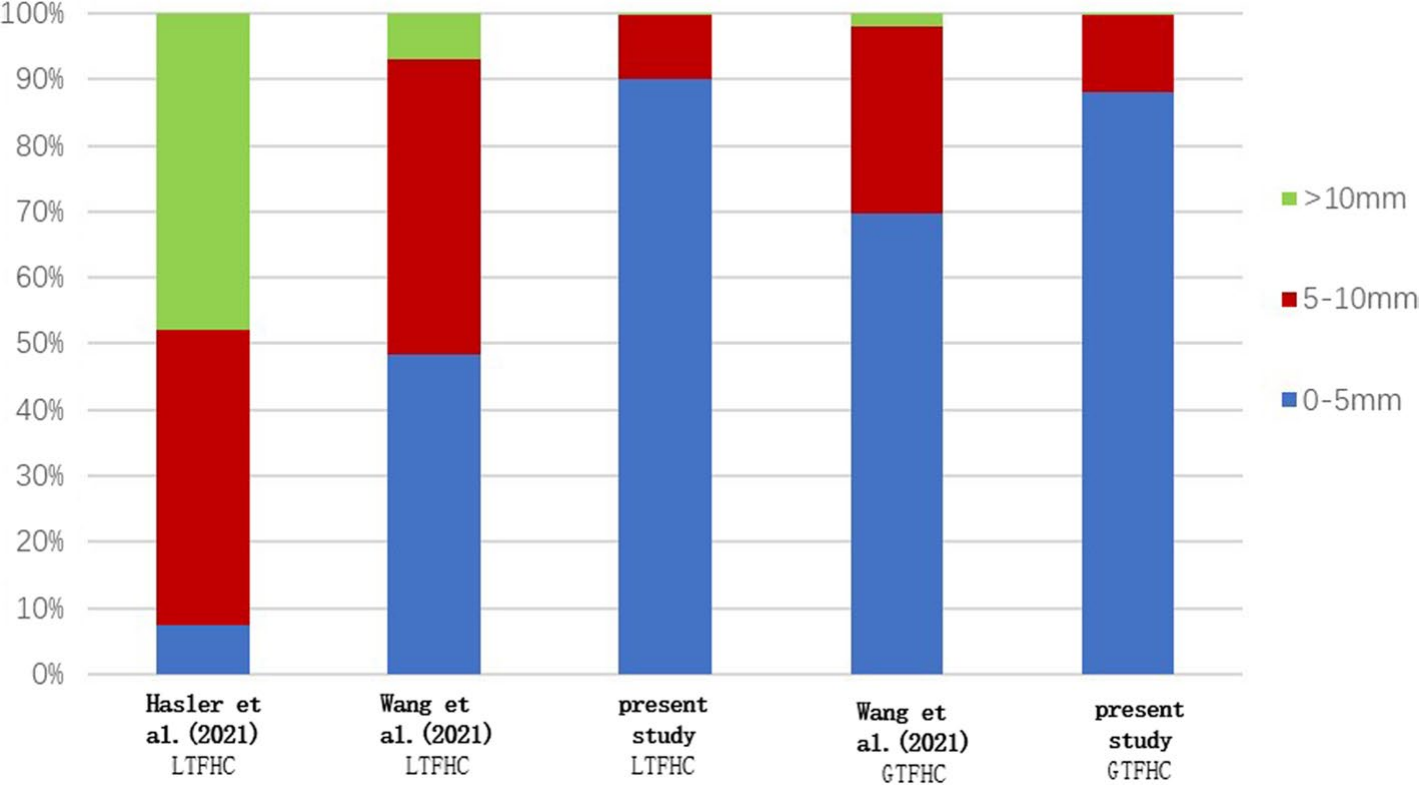
Frequencies of categorized differences between predicted and measured values of LTFHC and GTFHC applying the ratios described by different authors on the total study population (*n* = 294)

**Fig. 4:**
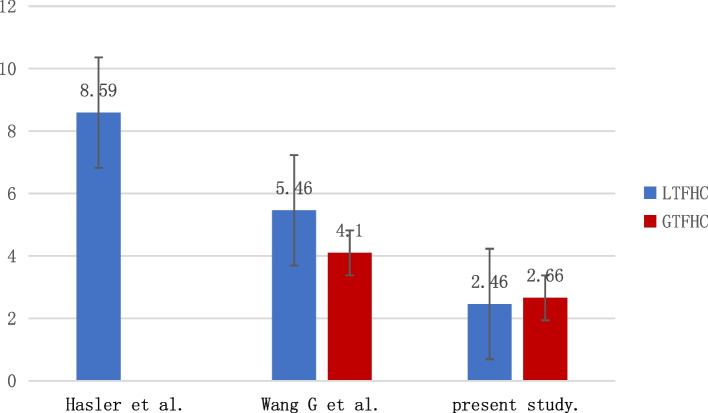
Standard deviation of the differences between predicted and measured values of of LTFHC and GTFHC applying the ratios described by different authors on the total study population (*n* = 294)

We compared our study with other studies regarding inclusion population, measurement technique, sample size, ratio, and error values (Tables 4 and 5; Figs. 3 and 4). Applying the Hasler et al. rate to the study population, the mean difference in LTFHC was 8.59 mm. Applying the rates of Wang (2021) to the study population, the mean differences in LTFHC and GTFHC were 5.46 and 4.10 mm, respectively. Using the ratios proposed in this study, the mean differences in LTFHC and GTFHC were 2.46 mm and 2.66 mm, respectively, as summarised in Table 5.

**Table 4:** Overview of Literature Regarding the LTFHC and GTFHC

Author + year	Unnanuntana [12] (2010)	Polishchuk [8] (2013)	Hasler [10] (2021)	WangGD [11] (2021)	Present study
Base population	USA	USA	USA	China	China
Number of cases	200	100	50	97	293
Technique	Digital photographs	X-rays	3D CT scans	X-rays	3D CT scans
Research Parameters	LTFHC	LTFHC	LTFHC	LTFHC GTFHC	LTFHC GTFHC

**Table 5:** Differences in ratios and estimated values between this study and the previous literature

Author + year	LTFHC/ FHD	LTFHC predicted	Absolute LTFHC Difference	GTFHC/ FHD	GTFHC predicted	Absolute GTFHC difference
Hasler et al. (2021) [10]	1.16	53.66± 4.60	8.59± 2.75	-	-	-
Wang et al. (2020) [11]	0.84	42.66± 4.21	5.46 ± 2.97	0.92	43.48± 3.13	4.10± 4.76
This study	Male0.94 Female0.96	44.88± 3.53	2.46± 1.94	Male:0.98 Female:1.00	46.75 ± 3.04	2.66 ± 1.96

Estimates were calculated using femoral head diameter (FHD) (measured in this study) and ratios (obtained in previous literature)

## Discussion

The improper rotation of the femoral head center is one of the typical complications observed with hip arthroplasty [13, 14], which often results in changes to hip biomechanics, gait abnormalities, lower back pain, sciatica, joint instability, and an increased risk of dislocation [2, 15], thus adversely affecting the subjective perception of the patient and the recovery and restoration of clinical function after hip arthroplasty. As a result, orthopedic surgeons are constantly investigating methods to restore the proper position of the FHC. Kumar et al. [16] found that placing the same vertical distance from the FHC to the apex of the greater trochanter on the healthy side could restore the HJC [16]. However, due to the limited exposure of the proximal femur, it is difficult to determine the vertical orientation during surgery.

Different authors have demonstrated that LTFHC and GTFHC can be used as parameters for the recovery of the FHC during surgery. Polishchuk et al. investigated the effects of demographic parameters such as sex, race, weight, and age on the parameter LTFHC [17]. They developed a formula based on multivariate regression that was somewhat complicated. Hasler et al.[18]found a mean ratio of 1.16 between LTFHC and FHD by measuring 3D CT data in the Western population [18]. Wang et al. analyzed the results of 100 Chinese adult pelvic orthopantomographs. They found that the ratio of LTFHC to FHD was approximately 0.84, and the ratio of GTFHC to FHD was approximately 0.92 [11].

We found that the mean ratios of GTFHC/FHD and LTFHC/FHD in the Chinese population, as measured by 3D CT scanning, were 0.99 and 0.95, respectively. These ratios are slightly higher than the value measured by Wang et al. likely because our measurements were based on 3D CT imaging. In contrast, the measurements of Wang et al. were based on 2D images (anteroposterior radiographs) *and* are susceptible to projection errors in the 3D geometry of the proximal femur [19, 20]. Haleret et al. measured femur models in Western populations by 3D CT imaging and recorded results with relatively higher ratios than this study, suggesting a difference between Chinese and Western populations.

The surgeon’s assessment of soft tissue tension during hip arthroplasty in patients determines leg length after placement of a trial prosthesis [21]. On the other hand, soft tissue tension is affected by various factors, including muscle mass and the amount of anesthetic utilized [22]. The FHD is the anatomical parameter of the proximal femur that is unaffected by a femoral neck fracture. In hip arthroplasty for patients with femoral neck fractures, the FHD is not altered by the femoral neck fracture. By multiplying the FHD with a particular ratio, LTFHC and GTFHC can be determined.

Although preoperative planning of hip replacement using the contralateral side is common [23–25], the lateral discrepancies in the 3-dimensional geometry of the proximal femur may generate inaccuracies in various template approaches [26–28]. There are limited data on the lateral variability of LTFHC and GTFHC [18]. Understanding the normal variation in these parameters may improve the accuracy of preoperative planning for hip arthroplasty. Our study found that the absolute LTFHC lateral difference in our population was 1.41 ± 1.18 mm, the mean LTFHC lateral difference was 0.09 ± 1.83 mm with a correlation coefficient of *r* = 0.90 (*P* < ;0.01).The mean absolute value of the lateral difference for GTFHC was 1.81 ± 1.41 mm, the mean GTFHC lateral difference was 0.17 ± 2.29 mm with a correlation coefficient of *r* = 0.861 (*p* < 0.01). Overall, 96% of LTFHC had an absolute lateral difference < 5 mm, 94% of the GTFHC had absolute lateral differences of < 5 mm. The differences between the two sides were small. They may not produce clinical symptoms because of the resulting template, and most smaller LLD or FO inequalities after hip arthroplasty have no or few symptoms. Therefore, the healthy-side LTFHC and GTFHC are reliable reference parameters for preoperative template and intraoperative validation of hip arthroplasty.

Sarint et al. [29] emphasized the importance of pelvic body position in measuring the FO distance and bilateral lower limb length [29]. In this study,the GTFHC and the LTFHC,were selected as the parameters for restoring FHC, which theoretically reduced the measurement error caused by the difference between the femoral and pelvic body positions.

The work in this study represents the first time that the parameter GTFHC has been investigated in 3 dimensions. It is also the first time the parameter LTFHC has been investigated in a sufficiently large sample of the Chinese population.We analyzed the the relative position of the femoral head center, greater trochanter, and lesser trochanter based on 3D-CT image reconstruction.. These data could provide a reference for restoring FHC during hip arthroplasty for hip fracture, especially in the absence of a contralateral hip reference.

However, this study does have limitations:

- 1. Articular cartilage was not included in creating the 3D model of the femur because a CT scan was used. The thickness of the femoral cartilage varies from patient to patient. There may be an effect on the femoral head diameter measurement in the results.In further studies, it may be more accurate to measure the results using MRI scan data.
- 2. The position of the LT on the proximal femur’s surface elevation is relatively unaffected by the anatomy of the proximal femoral diaphysis and the end of the diaphysis. It is positioned medial to the femoral stem from almost posterior to distally superior [30]. Some inaccuracies are expected due to the anterior, posterior, and medial portions of the lesser trochanteric position to the LTFHC distance.
- 3. The greater trochanter’s shape is quite variable [31], and the introduction of some measurement errors is anticipated. Based on the relative position of the apex of the greater trochanter to the median axis of the femoral medullary,the greater trochanter can be classified into five types:: anterior leaning, posterior leaning, centred and flat. A few greater trochanters had an additional, far-anterior protrusion that resulted in a false double apical appearance.The anterior leaning, posterior leaning and centred greater trochanter apexes are easier to identify and account for more than 80% of cases [31].For the flat and false double apical types, we did not measure because the greater trochanteric apex was not easy to confirm.
- 4. There are only Chinese hip data available. As a result, a cross-sectional investigation using 3D CT scans of asymptomatic persons of various races will be required. As Japanese and Koreans have comparable body types to Chinese people, the findings of this study may be useful for patients and researchers in these nations.

## Conclusion

We conclude that the greater trochanter and the lesser trochanter can be used as reliable landmarks to precisely locate the FHC. LTFHC and GTFHC are reliable reference parameters for preoperative template and intraoperative verification of hip arthroplasty.The ratio displayed in this research may provide a practical and simple method for orthopedic surgeons to perform hip arthroplasty in patients with femoral neck fractures. Furthermore, ratios from studies based on the same race may be preferable. These data could provide a reference for restoring FHC during hip arthroplasty for hip fracture, especially in the absence of a contralateral hip reference.

LTFHC and GTFHC are reliable reference parameters for preoperative planning and reconstruction of FHC of hip arthroplasty.

## Data Availability

The datasets generated and/or analyzed during the current study are not publicly available due to limitations of ethical approval involving the patient data and anonymity but are available from the corresponding author on reasonable request.
